# Medical, pharmacy and nursing students in the Baltic countries: interactions with the pharmaceutical and medical device industries

**DOI:** 10.1186/s12909-020-02008-5

**Published:** 2020-04-06

**Authors:** Ieva Salmane-Kulikovska, Elita Poplavska, Signe Mezinska, Vita Dumpe, Helena Dauvarte, Lina Lazdina, Aleksandr Marchockij, Karolis Varzinskas, Barbara J. Mintzes

**Affiliations:** 1 Faculty of Pharmacy, Riga Stradins University, Dzirciema street 16, Riga, LV 1007 Latvia; 2 Faculty of Pharmacy and Institute of Public Health, Riga Stradins University, Dzirciema street 16, Riga, LV 1007 Latvia; 3 Faculty of Medicine and Institute of Clinical and Preventive medicine, University of Latvia, Raina blvd. 19, Riga, LV-1050 Latvia; 4 Health Projects for Latvia, Baznicas street 5 - 2, Riga, LV 1010 Latvia; 5 Riga Stradins University, Dzirciema street 16, Riga, LV 1007 Latvia; 6 Pauls Stradins Clinical University Hospital, Pilsoņu street 13, Riga, LV-1002 Latvia; 7 Lithuanian University of Health Sciences, A. Mickevičiaus g. 9, Kaunas, Lithuania; 8 Faculty of Pharmacy and Charles Perkins Centre, The University of Sydney, Room 6W75, 6th Floor, The Hub, Charles Perkins Centre D17, Sydney, NSW 2006 Australia

**Keywords:** Medical students, Pharmacy students, Nursing students, Pharmaceutical industry, Medical device industry, Education, Latvia, Lithuania, Estonia

## Abstract

**Background:**

Interactions between pharmaceutical and medical device industries and students can lead to commercial influences on educational messages, with a potential to bias future treatment choice. This is the first study in the Baltic countries describing exposure and attitudes of medical, pharmacy and nursing students towards cooperation with industry.

**Methods:**

A cross-sectional on-line survey of current medical, pharmacy and nursing students (*n* = 918) in three Baltic countries was carried out.

**Results:**

We found that most students participate in events organized or sponsored by industry and accept a range of gifts and benefits. Students in the Baltic countries consider cooperation with industry important; at the same time, most do not feel that they have sufficient training on how to ethically interact with pharmaceutical and medical device companies and believe that these interactions can influence their prescribing or dispensing patterns. There is a tendency to rationalize cooperation with industry by referring to the current economic situation and patient benefits. Pharmacy students have higher rates of participation and they accept gifts and other benefits more often than nursing or medical students; therefore, they are likely to be more vulnerable to potential industry influence.

**Conclusions:**

The findings highlight the need to include topics on ethics and conflicts of interests in cooperation with industry in curriculum of health care students in Baltic countries. Without proper training, students continue to be at risk to industry influence and may develop habits for their further practice differing from evidence-based practice in prescribing and dispensing of medicines, as well as use of medical devices.

## Background

A range of studies in different countries describing relations between pharmaceutical and medical device industry and specialists have found that physicians, pharmacists and nurses are usually first exposed to promotional interactions with the pharmaceutical and medical device industries during their studies [1, 2]. There is evidence that interactions between physicians and the pharmaceutical industry can lead to poorer quality prescribing [3–5]. For example, it has been found that industry sponsored continuing medical education is associated with increased prescription rates of the sponsor’s medicines [6, 7]. Student-industry relationships raise specific concerns, as without explicit policies regulating this relationship, regular interactions, acceptance of gifts and other material benefits becomes normalised [8].

A recently published study examined interactions between nurses and industry and found that they are similar to those of physicians [9]. Another study has compared attitudes and exposure of pharmacists and physicians, finding that physicians had more exposure to interactions with the industry, but more pharmacists had received training on drug promotion [10]. There were differences in perceived usefulness of promotional benefits – pharmacists valued information on new drugs and free drug samples, whereas physicians – conference registration fees and free drug samples [10]. To the authors’ knowledge, there are no studies examining attitudes and exposures of medical, nursing and pharmacy students altogether.

The Baltic countries (Estonia, Latvia, Lithuania) underwent a rapid transition to a market economy with the dissolution of the Soviet Union in the early 1990s and all joined the European Union in 2004. The healthcare systems in all three Baltic countries were severly affected by the global economic crisis of 2008 and have been gradually recovering since then. However, healthcare expenditure in 2016 in all three countries was low, compared to other countries belonging to the Organisation for Economic Co-operation and Development (OECD). In Estonia, 6.7%, in Latvia 6.2% and in Lithuania 6.7% of gross domestic product (GDP) is devoted to healthcare financing [11]. Lack of financing has impact also on healthcare professionals’ practices and educational activities.

The European Federation of Pharmaceutical Industry Associations (EFPIA) introduced requirements for member companies of all European industry associations, including those in the Baltic countries, to disclose payments to health professionals through the EFPIA Disclosure code, also called the “European Sunshine Act” [12]. The EFPIA Disclosure code includes payments to physicians, nurses, pharmacists, other healthcare professionals, hospitals and healthcare organizations. These disclosure requirements affect health professional students as well as licensed practitioners. The current study provides important background information relevant to this policy initiative.

There are also no studies to date of interactions between the pharmaceutical and medical device industries and health professional students in the Baltic countries, and few studies of health professional-industry interactions in Eastern Europe or in the ex Soviet States. The objective of this study was to measure and compare medical, pharmacy and nursing students’ exposure to and attitudes about interactions with the pharmaceutical and medical device industry (further referred to as “industry”). The sample of the study enclosed students from all three Baltic countries.

## Methods

### Study design

This was an observational cross-sectional study. We conducted an anonymous online survey of medical, pharmacy and nursing students in Baltic countries in December 2016 to describe their exposure and attitudes towards interactions with the industry.

### Setting and participants

Respondents were recruited through students’ associations in all three Baltic countries. The survey link to the online questionnaire was sent via online survey tool *SurveyMonkey* to the previously approached representatives of the associations who further contacted their members and other students with valid e-mail addresses from the programmes Medicine, Pharmacy and Nursing in December 2016. Survey was sent to 4700 students in all three Baltic countries, and 918 responses were received. The online survey was self-administered and anonymous, in Latvian, Estonian and Lithuanian languages. To increase participation rates, as an incentive, respondents from each country could participate in a raffle to win 5–7 pairs of cinema tickets. E-mail addresses that were provided by raffle participants were automatically delinked from survey data to ensure anonymity. All students in medicine, pharmacy and nursing, at any study year, were invited to participate. Graduates were excluded from the survey.

### The survey measurement

The development of the survey questionnaire was based on a literature review and analysis of instruments used in similar surveys in other countries [13, 14] and expert consultations. Fourteen medical, pharmacy and nursing students from Latvia and Estonia took part in cognitive testing [15] of the English version of the questionnaire. Students were asked to evaluate quality (choice of words, length of the questions, mistakes and bias of the content), comprehension (clarity, whether there are additional explanations needed) and cultural sensitivity (whether a question causes embarrassment or is viewed negatively) of the questionnaire. Overall, quality, comprehension and cultural sensitivity of the questions were rated as acceptable. Wording of eight questions was adjusted for clarity. The final version of the survey questionnaire was then translated into Latvian, Estonian and Lithuanian by professional translators and reviewed by student representatives in the respective countries.

The questionnaire (40 questions, from which 36 were closed-ended and 4 - open-ended), included information on (1) demographics (gender, age, country) study programme, study year, employment status - 6 questions; (2) the respondent’s exposure to the industry, including participation in events organized or sponsored by industry, receipt of gifts (books, souvenirs, gifts, financial support, etc.) – 21 questions; and (3) attitudes towards interactions with the industry – 13 questions.

Students’ attitudes towards interactions with industry were measured both with statements suggesting positive attitude towards cooperation with industry, and statements suggesting scepticism towards cooperation with industry. Questions on exposure to industry activities and incentives were divided into two groups, one with five questions about attending sponsored events, (e.g. educational events, presentations by sales representatives, dinners, etc.), and the second with nine questions about receiving different items from the industry (e.g. lunch or food, gifts, educational materials, drug samples, etc.). The full text of the questionnaire is included in Additional file 1.

### Statistical methods

We used Chi-square tests (x^2^) to assess associations between exposure and attitudes and to assess whether observed frequencies per cell were greater than expected. To normalize the data in Chi-square hypothesis testing, we used the standardized residuals ratio.

Binary logistic regression analysis was used to test predictors of participation in the events and receipt of material benefits from industry. Two logistic regression models tested factors associated with 1) participation in industry-sponsored events; and 2) receipt of benefits from industry. Explanatory variables (predictors) entered into the model included gender, age, study programme (medicine, nursing or pharmacy), year of study, the country and attitudes suggesting acceptance or scepticism. *P* < 0.05 was considered statistically significant. All analyses were carried out using Microsoft SPSSv25.

All answers to open ended questions were coded by two researchers into categories that correspond to the main themes of the questionnaire.

### Ethical approval

According to the national regulations in Latvia and Code of Ethics of the Latvian Sociological Association [16] research ethics committee review is not required for anonymous sociological surveys. In the introductory part of the anonymous online questionnaire all participants were informed in writing about the aim of the study, contact information of the researchers, rights of the research participants and voluntariness of the participation. Consent to participate in the study was implied by the completion and submission of the form.

## Results

### Study population

We received 920 questionnaire responses, 2 of which were excluded because the respondents had already graduated. The total number of cases included in the analysis was 918, 223 (24.3%) of whom were respondents from Latvia, 389 (42.4%) from Lithuania, and 306 (33.3%) from Estonia. In total, 646 (70.4%) were medical students, 148 (16.1%) were pharmacy students, and 124 (13.5%) - nursing students. Demographic characteristics are displayed in the Table 1.

**Table 1:** Demographic characteristics of the respondents

Country	Latvia (*n* = 223)	Lithuania (*n* = 389)	Estonia (*n* = 306)	Total (*n* = 918)
Mean age (SD)	23.3 (SD 3.7)	21.3 (SD 2.1)	23.1 (SD 3.6)	22.4 (SD 3.2)
Age range	18–49	18–33	19–43	18–49
Women, %	89.2	78.9	78.8	81.4
Men, %	10.8	21.1	21.2	18.6
Medical students^a^, %	65.9	74.8	68.0	70.4
Pharmacy students^a^, %	18.4	16.5	14.0	16.1
Nursing students^a^, %	15.7	8.7	18.0	13.5
Undergraduate students^a^ (years 1–6), %	97.7	99.7	100.0	99.4
Residency in medicine^a^, %	1.4	0.3	–	0.4
Post-graduate studies (for pharmacy and nursing students)^a^, %	0.9	–	–	0.2
Working during their studies^a^, %	57.0	26.2	36.9	37.7

^a^from all respondents within a country

As there were minor differences between three countries regarding exposure and attitudes, for the further analysis, we combined the countries to explore differences between professions.

### Exposure to industry activities and incentives

#### Participation in events, sponsored or organized by industry

In general, 66.2% of the students in all three countries had participated at least once in an event that was financed or organized by industry. These included educational, informative or social events. During the last year, 78.3% took part in such events 1–5 times and 2.3% participated 6 times or more (19.4% did not specify frequency of participation).

Among those students who reported participation in sponsored events, 52.8% stated that they were informed that the event was sponsored or financed by industry, 30.0% claimed that they were not informed, but 17.2% chose the answer “I do not know” or did not respond.

As it is shown in Table 2, 39.8% of students in all three Baltic countries had participated in educational events including a lecture by sales representatives outside the study course. Overall, 35.7% of the respondents from all three countries had taken part in conferences or meetings for healthcare providers organized or sponsored by industry and 30.7% had participated in industry-sponsored social event. Some examples of social events mentioned by respondents included sports events, Christmas parties, recreational events, markets, concerts, dinner, etc. 30.3% of the respondents in all three countries reported having listened to presentations from sales representatives about new drugs and devices, and 26.8% claimed that they had attended educational events as a part of the university course.

**Table 2:** Participation in the events and receipt of benefits from industry, %

Participation in the events, organized or sponsored by industry				
	Medicine	Pharmacy	Nursing	Total
Educational event aimed at students with a lecture from sales representatives, outside the study course	39.3 (218/554)	**50.8** (67/132)	**29.4** (32/109)	39.8 (317/796)
Conferences or meetings for healthcare providers, sponsored or organized by industry	37.0 (205/554)	42.1 (56/133)	**21.3** (23/108)	35.7 (284/795)
An industry-sponsored social event (dinner, sports event, etc.)	29.7 (161/542)	**46.1** (59/128)	**17.1** (18/105)	30.7 (238/775)
Presentations by sales representatives	**27.8** (161/580)	**44.6** (58/130)	27.0 (30/111)	30.3 (249/821)
Educational event as a part of the university course	**20.1** (109/542)	**52.7** (68/129)	29.5 (33/112)	26.8 (210/783)
**Receipt of food, gifts, sponsorship, etc. from industry**				
	**Medicine**	**Pharmacy**	**Nursing**	**Total**
Brochures, journals, magazines	**56.6** (307/542)	**76.0** (92/121)	55.1 (54/98)	59.5 (453/761)
Small gifts (value less than 10 EUR)	49.9 (271/543)	**66.1** (80/121)	**42.1** (40/95)	51.5 (391/759)
Free lunch, food, coffee, sweets, etc.	**27.6** (144/522)	**57.1** (68/119)	25.3 (24/95)	32.1 (236/736)
Drug samples	**21.8** (119/545)	**43.3** (52/120)	**10.2** (10/98)	23.7 (181/763)
Text book(s)	10.6 (57/540)	10.0 (12/120)	10.3 (10/97)	10.4 (79/757)
Gift of value more than 10 EUR	**5.2** (28/537)	**13.3** (16/120)	4.2 (4/95)	6.4 (48/752)
Medical device or instrument	4.4 (24/543)	1.6 (2/123)	5.1 (5/98)	4.1 (31/764)
Financial support for attending conferences	2.8 (15/541)	5.9 (7/119)	2.1 (2/97)	3.2 (24/757)
**Felt peer pressure**				
	**Medicine**	**Pharmacy**	**Nursing**	**Total**
To participate in events	5.7 (30/530)	**16.5** (19/115)	12.5 (12/96)	8.2 (61/741)
To accept benefits	4.3 (23/541)	5.1 (6/118)	5.3 (5/95)	4.5 (34/754)

Bolded text refers to responses that differ significantly from the total (adjusted standardized residuals value > 1.96 or < −1.96) at *p* < .05.

In paranteses, the number of students who gave positive answer and the total number of those who answered the question is presented. Answers “I do not wish to answer” were excluded from the analysis

Of the three professions, pharmacy students had the highest participation rates in industry-sponsored events (Table 2). In answers to open ended questions, students from all three countries noted that participation in the events sponsored or organized by industry was beneficial, and if they had known about these events, they would have taken part in them more often. As stated by a student in Lithuania in an answer to open ended question: *“I would participate more, if I knew where such conferences take place”*.

#### Receiving gifts or other material benefits from industry

Overall, within last year, 60.2% of the respondents had received some material benefits from industry; 69.8% received benefits 1–5 times and 4.0% received benefits more than 6 times (26.2% did not specify frequency of receiving benefits). In their comments, students mentioned different types of benefits or gifts, such as free drug samples, pens, stethoscopes, notebooks, calendars, toothbrushes, etc.

In the three countries combined, 59.5% of students reported having received brochures and magazines from sales representatives (see Table 2). 51.5% accepted small gifts (under 10 EUR). 32.1% reported having received free lunch, food, coffee, sweets, etc., 23.7% - free drug samples, 10.4% had received text books from sales representatives, and 6.4% gifts worth over 10 EUR. In addition, 4.1% had accepted medical devices or instruments and 3.2% financial support to attend conferences.

Pharmacy students had higher rates of receiving brochures, journals and magazines, gifts, free lunch and drug samples. Several students commented they believe that financial support is useful for students, as scholarships are low: “*Sales representatives come so often and give many things (pens, etc.), so I perceive it as a normal thing. I am even expecting these gifts”* (Latvian student). Attitudes towards small gifts, such as pens and free drug samples, differed from more expensive gifts: “*Gifts, such as pens or sample products, should not be condemned as everybody is handing them out. I think that bigger gifts will start to influence our decisions, though subconsciously”* (Estonian student). Around one fifth of students have received drug samples. An Estonian student commented that “*free drug samples are like a gift from heaven for a poor Estonian – especially in family medicine”.*

In total, 46.7% of respondents reported that their professors use stationery and educational materials (notebooks, pens, books, etc.) with industry logos or advertisements. Students also mentioned that some of their textbooks contain medicines advertising.

### Peer pressure

We asked about peer pressure to participate in events and accept benefits from industry (Table 2). More pharmacy students (16.5%) reported pressure to participate in events organized or sponsored by industry than nursing or medical students.

### Attitudes towards interactions with industry

For the purpose of analysis, questions about attitudes towards interactions with industry were divided into two groups: acceptance and scepticism [2]. Tables 3 and 4 describe these results.

**Table 3:** Positive attitudes towards cooperation with industry, %

	Agreement with statement			
Statement	Medicine	Pharmacy	Nursing	Total
It is important for students to attend educational events organized or sponsored by industry	**52.3** (243/465)	**84.4** (92/109)	67.4 (58/86)	59.5 (393/660)
Industry should participate in the education of students at universities	**32.9** (153/465)	**65.5** (72/110)	**60.5** (52/86)	41.9 (277/661)
Universities should promote financial collaboration between industry and faculty	**26.7** (121/453)	**66.3** (69/104)	**57.0** (45/79)	36.9 (235/636)
Industry provides objective, reliable and high-quality information about medicines and medical devices	**30.2** (132/437)	**53.8** (57/106)	43.2 (32/74)	35.8 (221/617)
I have enough information/knowledge on how to ethically interact with industry	**30.8** (136/441)	39.8 (39/98)	39.2 (31/79)	33.3 (206/618)
Industry should be seen as equal partner in the health care system along with patients, health care providers and government institutions	**29.8** (128/429)	37.5 (36/96)	39.2 (29/74)	32.2 (193/599)

Bolded text refers to responses that differ significantly from the total (adjusted standardized residuals value > 1.96 or < − 1.96).

In paranteses, the number of students who gave positive answer and the total number of those who answered the question is presented. Answers “I do not wish to answer” were excluded from the analysis

**Table 4:** Attitudes suggesting scepticism, %

	Agreement with statement			
Statement	Medicine	Pharmacy	Nursing	Total
Students should receive training about ethical aspects of interactions between health care providers and industry	89.1 (443/497)	91.2 (103/113)	88.0 (79/89)	89.4 (625/699)
Free drug samples are a marketing tool	74.8 (363/485)	65.2 (73/112)	69.8 (60/86)	72.6 (496/683)
Gifts, financial support (including travel grants for conferences, consultancy fees etc.) from industry can influence a professional’s prescribing/dispensing habits	71.1 (335/471)	61.6 (61/99)	65.0 (52/80)	68.9 (448/650)
If a lecturer received a gift, financial or intellectual support from industry, she/he must always disclose it to the audience	61.1 (286/468)	54.5 (54/99)	63.4 (52/82)	60.4 (392/649)
Gifts from industry to physicians, pharmacists and nurses must be limited	**35.0** (164/468)	23.8 (24/101)	**21.0** (17/81)	31.5 (205/650)
Gifts from industry to students must be limited	**31.5** (140/444)	**20.0** (20/100)	23.8 (19/80)	28.7 (179/624)
Funding from industry to the physicians, pharmacists and nurses must be limited	**17.2** (80/464)	**5.5** (6/109)	16.3 (14/86)	15.2 (100/659)
Funding from industry to students must be limited	11.6 (55/473)	5.7 (6/106)	13.4 (11/82)	10.9 (72/661)

Bolded text refers to responses that differ significantly from the total (adjusted standardized residuals value > 1.96 or < − 1.96).

In paranteses, the number of students who gave positive answer and the total number of those who answered the question is presented. Answers “I do not wish to answer” were excluded from the analysis

#### Attitudes suggesting acceptance of cooperation with industry

As is seen in Table 3, 59.5% of the students believed that it is important to attend educational events organized or sponsored by industry and 41.9% held the view that industry should participate in education. Pharmacy students were more accepting of interaction with industry than nursing or medical students. Students noted that financial support from industry was important to them to be able to participate in educational events, especially internationally. One Estonian student commented that *“at the moment there are no other alternatives*”.

#### Attitudes suggesting scepticism

As shown in Table 4, 89.4% of students considered that “students should receive training about ethical aspects of interactions between health care providers and industry”, and 72.6% agreed that free drug samples are a marketing tool. Students were aware that gifts and financial support from industry may influence a professional’s prescribing/dispensing habits – 68.9% agreed with this statement.

More than half of the students also believed that lecturers who received gifts, financial or intellectual support from industry should always disclose this. However, only 10.9% students agreed that funding from industry to students must be limited. As it is seen in Table 4, medical students were more likely than pharmacy and nursing students to believe that gifts and funding should be limited.

### Factors associated with participation in events and receipt of benefits

Educational level (study year) was a significant predictor of participation in sponsored events Table 5). For each additional year of studies, the likelihood of participation in sponsored events increased: odds ratio (OR) = 1.55 (95% CI 1.23–2.00; *p* < 0.001), as did the likelihood of receiving financial benefits: OR = 1.42 (95% CI 1.14–1.76; *p* = 0.002).

**Table 5:** Predictors of participation in events and receipt of benefits

Predictor	OR	*P*-value	95% CI
*Participation in events organized or sponsored by industry*			
Study year (per year of advancement)	1.55	0.001	1.23–2.00
*Receiving financial benefits*			
Gender - male	0.302	0.001	0.16–0.58
Study programme pharmacy *(*vs *medicine*)	4.96	0.007	1.54–16.0
Study year	1.42	0.002	1.14–1.76

Gender was also a significant predictor of receipt of financial benefits from industry, with male students less likely than female students to receive these benefits: OR = 0.302 (95% CI 0.16–0.58; p < 0.001). Pharmacy students were also more likely to receive financial benefits than medical students OR = 4.96 (95% CI 1.54–16.0, *p* = 0.007). The likelihood of receiving material benefits did not differ by country. Students’ attitudes and likelihood of having received financial benefits were not statistically significantly associated.

## Discussion

More than half of the respondents in all three Baltic countries had participated in events organized or sponsored by industry. Pharmacy students were more exposed to the events and also reported the highest peer pressure to attend events sponsored or organized by industry. Our study shows that each study year increased the likelihood of participation in the events organized or sponsored by industry - interactions with industry became more frequent as a student progressed thorough their studies. Also, other studies have noted that more experienced physicians and pharmacists are usually approached by industry more often [10].

More than half of the students in our study had received some material benefits during the previous year. Brochures, journals, magazines, as well as small gifts were the most frequently accepted benefits, and pharmacy students accepted benefits from industry more often than other groups of students. Our study also shows that pharmacy students had experienced more peer pressure to accept benefits and participate in different events organized or sponsored by industry. The higher exposure to interactions with the industry in the group of pharmacy students could be explained by the fact that these students are more likely to become employees of the industry in the future compared to other health care students. It might increase their motivation to interact with the industry during studies, as well as attempts of the industry to approach these students. Although pharmacists do not have a right to prescribe medicines, generic substitution is allowed in the pharmacies which also might explain interest of the pharmaceutical industry [17]. Reasons for differences in exposure might be sought by further research analyzing content of curricula and amount of professional training on ethical aspects of interactions with industry. Examining curricula in other countries, authors have different insights. For example, a study analysing curricula in 64 countries concluded that medical schools spend less time in education about medicines promotion compared to pharmacy schools [18]. However, examining situation in Kuwait authors observed that medical students have more extensive training in ethics of drug promotion than pharmacy students [19].

The comments from students showed that support from industry, including small gifts, was considered “useful and normal”, as students’ scholarships are low. Austad et al. in their study review [13] found that medical students were more approving of small gifts, justifying them having an educational purpose. It has also be noted that even small gifts have a powerful influence on a physician’s behaviour [20], because they create reciprocal obligation [21]. Only about one tenth of the students in our study agreed that support from industry to students must be limited.

The majority of the students considered that gifts, financial support (including travel grants for conferences, consultancy fees etc.) from industry could influence a professional’s prescribing/dispensing habits. The literature review by Norris et al. concluded that in most studies doctors deny that they are influenced by gifts [22]. The same attitude was revealed in a survey by Sierles et al. [2] and in a systematic review of medical students’ attitudes towards these interactions [13]. In contrast, students in our study have comparatively high awareness of the potential impact of cooperation with industry on their practices. Although our study did not reveal direct statistically significant relationship between attitudes and likelihood of having received financial benefits and attending events organized or sponsored by industry, it showed generally high level of acceptance of cooperation with industry and, at the same time, worries about potential impact of cooperation on prescribing or dispensing habits. Holding mutually contradictory views may cause cognitive dissonance, and this phenomenon is described, for instance, in the study by Chimonas et al. that revealed how physicians cope with the cognitive dissonance by using variety of denials and rationalizations of their behaviour, like emphasizing cooperation benefits, as well as benefits of needy patients [23]. In our study, students rationalized cooperation with industry by appealing to the current economic situation when scholarships of the students are low. Another way of rationalizing behaviour was emphasis on the benefits of needy patients who get free drug samples.

More than one third of the students thought that information provided by industry was objective and reliable. Studies of physicians have shown that physicians perceive information from industry to be comprehensive and accurate [24, 25] and often do not consult other sources to verify the information they have received from industry [26]. It has been suggested that physicians who rely on drug company information, prefer expensive brands, adopt newer medicines more quickly, show more inappropriate prescribing and write more prescriptions than their colleagues [27].

Only about one third of students considered that they have sufficient knowledge on how to ethically cooperate with industry. The research evidence has also demonstrated that physicians are not sufficiently prepared for interactions with sales representatives and critical evaluation of the information provided by them [22]. In our study, pharmacy students showed less sceptical attitudes. This differs from the results of a study in Saudi Arabia that examined the attitudes of both medical and pharmacy students and found that pharmacy students had more sceptical attitudes, perhaps reflecting the difference in cultural settings [10]. Other studies have pointed out that students need education and guidance on how to recognize conflicts of interests in interactions with industry, and particularly on the risks of reliance on materials provided by industry as an educational resource [28]. Most respondents in our study, similarly, stated that they felt they needed more education on interactions with industry.

### Study limitations

Due to chosen sampling method the results are not generalizable to all medical, pharmacy and nursing students in Baltic countries. Further studies are needed to be able to generalize the data and to disclose actual prevalence of students’ exposure and attitudes towards collaboration with industry. It is possible that the students may have underreported their associations with industry, and the reason of it may be social desirability bias [29]. Our respondents mostly represented undergraduates, and the opinions of resident and master students were underrepresented. However, this is the first study addressing medical, pharmacy and nursing students’ exposure and attitudes towards cooperation with industry in the Baltic countries, providing a valuable insight and tendencies.

## Conclusions

The findings of this survey show some worrisome tendencies – students are exposed to interactions with industry and rationalize it by appealing to the current economic situation and benefits of needy patients who get free drug samples. Cooperation with industry is important and convenient for students in the Baltic countries. At the same time, most of the students do not feel trained enough on how to ethically interact with industry and are aware of the fact this cooperation can have impact on their prescribing and dispensing patterns.

Pharmacy students may be at a comparatively higher risk, therefore guidance and education about ethical aspects of cooperation with industry should be at least equally essential for them as for medical students. Students of later years of study have higher likelihood of exposure. This highlights the need of amended curriculum on ethical aspects and conflicts of interests in cooperation with industry, focusing on later years of studies in particular. Without proper education, students continue to be at risk to industry influence and may develop habits for their further practice differing from evidence-based practice in prescribing and dispensing of medicines, as well as use of medical devices.

## Supplementary information

**Additional file 1.** Final version of questionnaire on exposure and attitudes of medical, pharmacy and nursing students towards cooperation with industry.

## Data Availability

The dataset generated and analysed during the current study is available in the OSF repository:

## Abbreviations

CI: Confidence interval
EFPIA: The European Federation of Pharmaceutical Industry Associations
GDP: Gross domestic product
OECD: The organisation for economic co-operation and development
OR: Odds ratio

## References

[1] Siddiqui UT, Shakoor A, Kiani S, Ali F, Sharif M, Kumar A (2014). Attitudes of medical students towards incentives offered by pharmaceutical companies- perspective from a developing nation- a cross sectional study. BMC Med Ethics.

[2] Sierles FS, Brodkey AC, Cleary LM, McCurdy FA, Mintz M, Frank J (2005). Medical students’ exposure to and attitudes about drug company interactions: a National Survey. JAMA.

[3] Robertson C, Rose S, Kesselheim AS. Effect of financial relationships on the behaviors of health care professionals: a review of the evidence. J Law Med Ethics. 2012;40(3):452–66. 10.1111/j.1748-720X.2012.00678.x 23061573

[4] Sondergaard J, Vach K, Kragstrup J, Andersen M (2009). Impact of pharmaceutical representative visits on GPs’ drug preferences. Fam Pract.

[5] Wazana A (2000). Physicians and the pharmaceutical industry - is a gift ever just a gift?. Jama-J Am Med Assoc.

[6] Jutel A, Menkes DB (2008). Soft targets: nurses and the pharmaceutical industry. PLoS Med.

[7] Bowman MA, Pearle DL (1988). Changes in drug prescribing patterns related to commercial company funding of continuing medical education. J Contin Educ Heal Prof.

[8] Scheffer P, Guy-Coichard C, Outh-Gauer D, Calet-Froissart Z, Boursier M, Mintzes B, et al. Conflict of Interest Policies at French Medical Schools: Starting from the Bottom. Wray KB, editor. PloS One. 2017;12(1):e0168258. 10.1371/journal.pone.0168258 PMC5221756 28068362

[9] Grundy Q, Bero LA, Malone RE (2016). Marketing and the Most trusted profession: the invisible interactions between registered nurses and industry. Ann Intern Med.

[10] Zaki NM (2014). Pharmacists’ and physicians’ perception and exposure to drug promotion: a Saudi study. Saudi Pharm J.

[11] OECD. Health expenditure and financing [Internet]. 2017 [cited 2019 Aug 23]. Available from: https://stats.oecd.org/Index.aspx? DataSetCode=SHA.

[12] EFPIA. Code on Disclosure of Transfers of Value form Pharmaceutical Companies to Healthcare Professionals and Healthcare Organisations.

[13] Austad KE, Avorn J, Kesselheim AS. Medical students' exposure to and attitudes about the pharmaceutical industry: a systematic review. PLoS Med. 2011;8(5):e1001037. 10.1371/journal.pmed.1001037 PMC3101205 21629685

[14] PASS. Pharmawarenss student survey. 2016 [cited 2019 Aug 23]. Available from: http://www.pharmaware.co.uk/pass1.html#.

[15] Willis DGB (2004). Cognitive interviewing: a tool for improving questionnaire design.

[16] Latvian Sociological Association. Code of Ethics. [Internet]. 2008. [cited 2020 March 6]. Available from: http://sociologija.ait.lv/?page_id=73.

[17] Kawalec P, Tesar T, Vostalova L, Draganic P, Manova M, Savova A, et al. Pharmaceutical Regulation in Central and Eastern European Countries: A Current Review. Front Pharmacol [Internet]. 2017 Dec 18 [cited 2019 Nov 19];8. Available from: https://www.ncbi.nlm.nih.gov/pmc/articles/PMC5741607/. 10.3389/fphar.2017.00892 PMC5741607 29326583

[18] Mintzes B. Educational initiatives for medical and pharmacy students about drug promotion: an international cross-sectional survey [internet]: WHO, HAI, EU; 2005. Available from: https://apps.who.int/medicinedocs/pdf/s8110e/s8110e.pdf.

[19] Ball DE, Al-Menea SA. Exposure and attitudes to pharmaceutical promotion among pharmacy and medical students in Kuwait. Pharm Educ [Internet]. 2007 [cited 2019 Nov 20];7(4). Available from: http://pharmacyeducation.fip.org/pharmacyeducation/article/view/133.

[20] Dana J, Loewenstein G (2003). A social science perspective on gifts to physicians from industry. JAMA.

[21] Oldani MJ (2004). Thick prescriptions: toward an interpretation of pharmaceutical sales practices. Med Anthropol Q.

[22] Norris P, Herxheimer A, Lexchin J, Mansfield P. Drug promotion: What We Know What We Have Yet to Learn. World Health Organization [Internet]. 2005 [cited 2019 Nov 20]. Available from: https://apps.who.int/iris/handle/10665/69177.

[23] Chimonas S, Brennan TA, Rothman DJ (2007). Physicians and drug representatives: exploring the dynamics of the relationship. J Gen Intern Med.

[24] Lieb K, Scheurich A (2014). Contact between doctors and the pharmaceutical industry, their perceptions, and the effects on prescribing habits. PLoS One.

[25] Mintzes B, Lexchin J, Sutherland JM, Beaulieu M-D, Wilkes MS, Durrieu G (2013). Pharmaceutical sales representatives and patient safety: a comparative prospective study of information quality in Canada, France and the United States. J Gen Intern Med.

[26] Prosser H, Walley T (2003). Understanding why GPs see pharmaceutical reprensentatives: a qualitative interview study. Br J Gen Pract.

[27] Lexchin J (1993). Interactions between physicians and the pharmaceutical industry: what does the literature say?. CMAJ.

[28] Soyk C, Pfefferkorn B, McBride P, Rieselback R. Medical student exposure to and attitudes about pharmaceutical companies.. Wis Med J. 2010;109(3):142–8. 20672554

[29] Fisher RJ (1993). Social desirability Bias and the validity of indirect questioning. J Consum Res.

