# Correction to: Medical, pharmacy and nursing students in the Baltic countries: interactions with the pharmaceutical and medical device industries

**DOI:** 10.1186/s12909-020-02044-1

**Published:** 2020-04-22

**Authors:** Ieva Salmane-Kulikovska, Elita Poplavska, Signe Mezinska, Vita Dumpe, Helena Dauvarte, Lina Lazdina, Aleksandr Marchockij, Karolis Varzinskas, Barbara J. Mintzes

**Affiliations:** 1 Faculty of Pharmacy, Riga Stradins University, Dzirciema street 16, Riga, LV 1007 Latvia; 2 Faculty of Pharmacy and Institute of Public Health, Riga Stradins University, Dzirciema street 16, Riga, LV 1007 Latvia; 3 Faculty of Salmane-Kulikovska et al. BMC Medical Education (2020) 20:105 Page 8 of 9 Medicine and Institute of Clinical and Preventive medicine, University of Latvia, Raina blvd. 19, Riga, LV-1050 Latvia; 4 Health Projects for Latvia, Baznicas street 5 - 2, Riga, LV 1010 Latvia; 5 Riga Stradins University, Dzirciema street 16, Riga, LV 1007 Latvia; 6 Pauls Stradins Clinical University Hospital, Pilsoņu street 13, Riga, LV-1002 Latvia; 7 Lithuanian University of Health Sciences, A. Mickevičiaus g. 9, Kaunas, Lithuania; 8 Faculty of Pharmacy and Charles Perkins Centre, The University of Sydney, Room 6W75, 6th Floor, The Hub, Charles Perkins Centre D17, Sydney, NSW 2006 Australia

**Correction to: BMC Med Educ**

**https://doi.org/10.1186/s12909-020-02008-5**

Following publication of the original article [1], we have been notified that the publication is missing a link to the data repository.

The publication is updated as follow:

Availability of data and materials

The dataset generated and analysed during the current study is available in the OSF repository: https://osf.io/r73hx
