# Supplementary material for: Medical, pharmacy and nursing students in the Baltic countries: interactions with the pharmaceutical and medical device industries
Source: BMC Med Educ. 2020 Apr 6;20:105. doi: 10.1186/s12909-020-02008-5 (PMC7137495; doi:10.1186/s12909-020-02008-5)
Supplement: Supplementary file 1 — Additional file 1. Final version of questionnaire on exposure and attitudes of medical, pharmacy and nursing students towards cooperation with industry. [file 12909_2020_2008_MOESM1_ESM.doc]

**Additional file 1.**

**Survey on student interaction with pharmaceutical and medical device industries in the study process**

Medical, pharmacy and nursing students are invited to participate in a survey on student interaction with pharmaceutical and medical device industries during the study process and students' attitudes towards collaboration with pharmaceutical and medical device industries.

This study is implemented by the Health Projects for Latvia with the support of Health Action International. Dissemination of the survey takes place in cooperation with LaMSA and the Student Council of the University of Latvia. Participation in the study is voluntary and anonymous. Filling in the questionnaire will not take more than 10 minutes.

Participants will be able to win one of the 7 movie ticket pairs if you fill in the email address after completing the survey!

If you have any questions, please write to veselibasprojekti@gmail.com

## I Demographic information

1. Study program:
2. Medicine
3. Pharmacy
4. Nursing
5. Year of study:
6. Age:
7. Gender:
8. Female
9. Male
10. Do not wish to answer
11. Do you currently work or volunteer in a hospital/ clinic/pharmacy?
12. Yes
13. No
14. Do not wish to answer
15. Country of study:
16. Estonia
17. Latvia
18. Lithuania

## II Exposure to drug and medical device company interactions.

This section will assess the experience of students regarding cooperation with pharmaceutical and medical device industries. Please read each question / statement and give your answer. If necessary, provide comments in blank spaces.

| **Have you attended any of the events described below?** | | | | |
| --- | --- | --- | --- | --- |
|  | **Yes** | **No** | **Do not know** | **Do not wish to answer** |
| 1. An educational event aimed for students with a lecture from a representative of a pharmaceutical/medical device company or sponsored or organized by a company outside of your university courses. |  |  |  |  |
| 1. A lecture or seminar by a representative of the pharmaceutical or medical device company as part of your university course. |  |  |  |  |
| 1. A seminar, meeting or conference for health care providers sponsored or co-organized by a pharmaceutical or medical device company. |  |  |  |  |
| 1. A presentation aimed to inform about new drugs or devices given by a drug rep in a hospital, clinic or pharmacy. |  |  |  |  |
| 1. A dinner or social event sponsored or organized by a pharmaceutical or medical device company. |  |  |  |  |

1. Were you always informed about the company’s involvement or sponsorship before the event/lecture/seminar?

a) Yes

b) No

c) Do not know

d) Do not wish to answer

e) I did not participate in any of these kinds of events

1. Please describe below any other activities/events which you have attended that had some involvement of pharmaceutical or medical device companies.
2. How often have you attended events that were organized or sponsored by a pharmaceutical or medical device company or where a representative of an industry gave a lecture within the last year?
3. 0
4. 1-5
5. 6-10
6. More than 10
7. Do your university instructors use educational materials (e.g. manekens, notebooks, pens, books etc) with logos or advertisements of pharmaceutical and medical device companies?
8. Yes
9. No
10. Do not know
11. Do not wish to answer

| **Have you as a future health care provider received any of the following items or support from a pharmaceutical or medical device company?** | | | | |
| --- | --- | --- | --- | --- |
|  | **Yes** | **No** | **Do not**  **know** | **Do not**  **wish to**  **answer** |
| 1. Lunch, snacks, tea, coffee, sweets etc. |  |  |  |  |
| 1. A small item – a pen, notebook, cup, souvenir etc. of the value **less than** EUR 10. |  |  |  |  |
| 1. An item with no educational or professional meaning of the value **more than** EUR 10. |  |  |  |  |
| 1. A textbook – printed or electronic. |  |  |  |  |
| 1. A medical device or instrument (e.g. stethoscope). |  |  |  |  |
| 1. Other educational materials (brochure, journal or magazine). |  |  |  |  |
| 1. A drug sample |  |  |  |  |
| 1. Financial support for a conference attendance and/or travel and/or accommodation expenses |  |  |  |  |
| 1. Have you ever experienced encouragement or pressure from your peers, colleagues or supervisors to attend an event organized or sponsored by a pharmaceutical/medical device company? |  |  |  |  |
| 1. Have you ever experienced encouragement or pressure from your peers, colleagues or supervisors to accept an item or support provided by a pharmaceutical/medical device company? |  |  |  |  |
| 1. Other items or services received (please describe): | | | | |

1. How often have you accepted any of the described items/support within the last year?
2. 0
3. 1-5
4. 6-10
5. More than 10

## III. Attitudes regarding interaction with pharmaceutical and medical device industries

This section will evaluate attitudes of students towards the involvement of pharmaceutical and medical device industries in the process of educating students and specialists. Please read each question / statement and give your answer. If necessary, provide comments in blank spaces.

Please rate each statement:

|  | **Strongly**  **Agree/**  **Agree** | **Neither**  **agree or**  **disagree** | **Disagree/**  **Strongly**  **disagree** | **Do not**  **know** | **Do not**  **wish to**  **answer** |
| --- | --- | --- | --- | --- | --- |
| 1. Pharmaceutical and medical device companies provide objective, reliable and high-quality information on medicines and medical devices. |  |  |  |  |  |
| 1. Pharmaceutical and medical device companies should be seen as equal partners in health care system along with patients, health care providers and government institutions. |  |  |  |  |  |
| 1. It is important for students to attend educational events sponsored by pharmaceutical and medical device companies. |  |  |  |  |  |
| 1. Gifts or financial support to students from pharmaceutical and medical device companies should be limited. |  |  |  |  |  |
| 1. Gifts or financial support to health care providers from pharmaceutical and medical device companies should be limited. |  |  |  |  |  |
| 1. Funding for university lecturers and professors from pharmaceutical and medical device companies should be limited. |  |  |  |  |  |
| 1. Pharmaceutical and medical device companies should participate in the education of students at universities. |  |  |  |  |  |
| 1. Universities should promote financial collaboration between pharmaceutical and medical device companies and faculty. |  |  |  |  |  |
| 1. If a lecturer received a gift, financial or intellectual support from a pharmaceutical or medical device company, she/he must always disclose it to the audience |  |  |  |  |  |
| 1. Gifts, financial support (including travel grants for conferences, consultancy fees etc.) from a pharmaceutical and medical device companies can influence a professional’s prescribing/dispensing habits. |  |  |  |  |  |
| 1. Free drug samples is a marketing tool. |  |  |  |  |  |
| 1. I have enough information/knowledge on how to ethically interact with pharmaceutical and medical device companies. |  |  |  |  |  |
| 1. Students should receive training about ethical aspects of interactions between health care providers and pharmaceutical and medical device companies. |  |  |  |  |  |
